# The Effect of Activation Method of Rubber on the Performance of Modified Asphalt Binder

**DOI:** 10.3390/ma13173679

**Published:** 2020-08-20

**Authors:** Juan Xie, Yongning Zhang, Yueming Yang, Yunlong Ma, Jing Li, Menglong Huang

**Affiliations:** 1 School of Traffic and Transportation Engineering, Changsha University of Science and Technology, Changsha 410114, China; zhangyongning@stu.csust.edu.cn (Y.Z.); mayunlong1108@126.com (Y.M.); lijing@stu.csust.edu.cn (J.L.); 2 National Engineering Laboratory of Highway Maintenance Technology, Changsha University of Science and Technology, Changsha 410114, China; 3 Foshan Transportation Science and Technology Co., Ltd., Foshan 528000, China; 1710030028@stu.csust.edu.cn; 4 Continental Reifen Deutschland GmbH, 30419 Hannover, Germany; Menglong.huang@conti.de

**Keywords:** modified asphalt binder, activation method, coating activated rubber, grafting activated rubber, polyamide, acrylamide, performance

## Abstract

Poor storage stability is a key problem restricting the rapid development and wide application of rubber-modified asphalt binder, and activation of rubber has shown good prospects to solve this problem. In this study, two activation methods, coating by polyamide 6 and grafting by acrylamide, were introduced to treat crumb rubber. Then the activated rubber was added to base asphalt binder to prepare modified asphalt binder. The chemical structure and morphology of rubber powder before and after activation and of asphalt binder before and after modification were characterized by Fourier transformation infrared (FTIR) spectroscopy and scanning electron microscopy (SEM). The conventional and rheological properties and storage stability were analyzed to reveal the influence of activation method on the performance of asphalt binder. The results showed that after being activated, the surface of the rubber is loose and rough. A chemical reaction did not occur during activation by polyamide but occurred during activation by acrylamide. The activation of the rubber effectively improved the high- and low-temperature performance, and the softening difference decreased by 79.8%. This is because the interaction between rubber and asphalt binder was enhanced through activation of rubber, and grafting activation had better effect due to the chemical reaction between the basic amide groups of acrylamide and acid groups of asphalt binder.

## 1. Introduction

As an important road material, petroleum asphalt binder has received widespread attention. Improving its comprehensive performance or functionalizing it by modification is the current research hotspot. Compound modification with rubber, attapulgite, columnar diatomite, and disk diatomite can improve the high-temperature rheological and aging resistance of asphalt binder [1]. Graphene oxide is a typical two-dimensional carbon-based nanomaterial that has many advantages, such as large specific surface area and high surface free energy value [2], and it can be used as a modifier to increase the anti-aging and intrinsic healing capability of asphalt binder [3]. Adding food waste crayfish shells to matrix asphalt binder can not only reduce environmental pollution, but also improve the high-temperature stability, rheology, and stiffness of asphalt binder [4]. Due to its good elasticity, polymers can improve the rutting resistance and anti-fatigue character of asphalt binders [5,6]. Styrene-butadiene-styrene (SBS) is one of the most used polymer modifiers in modified asphalt binder because it has strong interaction with maltene components and thus improves the elastic proportion of asphalt binder and reduces its fluidity [7].

The rubber powder obtained by crushing waste tires is also considered to be an asphalt binder modifier with good development prospects [8,9,10,11]. Firstly, the modification of asphalt binder with crumb rubber can reduce environmental pollution caused by used tires, which is in line with the development direction of energy conservation, emission reduction and recycling in the field of transportation infrastructure construction [12,13,14]. Secondly, it has been confirmed that asphalt binder modified with rubber powder has good elasticity and fatigue resistance [15,16,17]. However, due to the big difference of density, molecular structure and polarity between rubber powder and base asphalt binder, the compatibility between them is poor. Therefore, the modified asphalt binder is easy to segregate during storage [18], which is one of the key problems restricting the development of asphalt rubber [19].

Many research works have been carried out to improve the compatibility between rubber and asphalt binder and thus increase the storage stability of asphalt rubber, which can be divided into three categories. (1) Improve the preparation process. Pais, J. The digestion time was prolonged by [20] to 48 h at the shear speed of 250 to 300 rpm, and found that the dispersion of the crumb rubber in the asphalt binder increased as the digestion time extended, the rheological properties of modified asphalt binder tended to be stable. But the storage stability without stirring was not mentioned. Terminal blend (TB) rubberized binder has attracted increasing attention due to its good workability and storage stability [21]. It is generally prepared with finer crumb rubber at high mixing temperature, high pressure and high shear rate [22]. By contrast with the ordinary asphalt rubber prepared by a wet process, the modification mechanism of TB rubberized binder is the degradation and desulfurization of rubber, which decreases the high temperature performance and prevents it from being used alone [23]. (2) Add stabilizer or compatibilizer. Trans-polyoctenamer rubber (TOR) is a typical reactive modifier and can promote the distribution of rubber particles in asphalt binder, and thereby improve the high temperature and storage stability [24,25]. Meanwhile, sulfur, high-density polyethylene (HDPE) and low-density polyethylene (LDPE) can also be used as a stabilizer to improve the storage stability of rubber asphalt binder [26,27,28]. However, the addition of stabilizer makes the modification mechanism more complicated and preparation process more difficult to control. (3) Pretreat crumb rubber. Microwave treatment can provide uniform volumetric heat and lead to the cleavage of disulfide bonds in rubber. Asphalt binder modified with treated rubber has better storage stability and viscoelasticity and lower temperature susceptibility than asphalt binder modified with untreated rubber [29]. Kabir, S.F. and coworkers activated the surface of rubber by a hybrid method combining microwave irradiation and bio-chemical treatment with five bio-oils, and then added the activated rubber to prepare modified asphalt binder. The results showed that the activation of rubber enhanced its interaction with asphalt binder and the storage stability of asphalt rubber was improved [30]. In additional, activation of rubber by hydrogen peroxide (H_2_O_2_) solution [31] and plasma technology [32] are also effective way to improve the storage stability of modified asphalt binder. At present, research on improving storage stability through the activation of rubber powder mainly focuses on the evaluation of the macroscopic performance of asphalt binder, but the research on the microscopic performance is insufficient.

In this paper, crumb rubber was coating activated with polyamide 6 and grafting activated with acrylamide, respectively. Three types of modified asphalt binder were prepared with activated and non-activated rubber. The chemical structure of rubber asphalt binder was characterized by Fourier transformation infrared (FTIR) spectroscopy, and their morphology was observed by scanning electron microscopy (SEM). To investigate the effect of activation method of rubber on the performance of modified asphalt binder, the conventional properties, rheology and storage stability were examined. The modification mechanism was explored by combining macro performance and micro structure.

## 2. Materials and Methods

### 2.1. Materials 

Base asphalt binder of grade 90 was provided by Heilongjiang Changhe Chemical Co., Ltd. (Suihua, China) and crumb rubber with a fineness of 60 mesh was purchased from Hengshui Zehao Chemical Company (Hengshui, China). Polyamide 6, acrylamide, acetone, formic acid and potassium persulfate were chemical grade and obtained from Sinopharm Chemical Reagent Co., Ltd. (Beijing, China). The technical parameters of base asphalt binder and crumb rubber are listed in Table 1 and Table 2.

### 2.2. The Activation of Crumb Rubber

**Figure 1:**
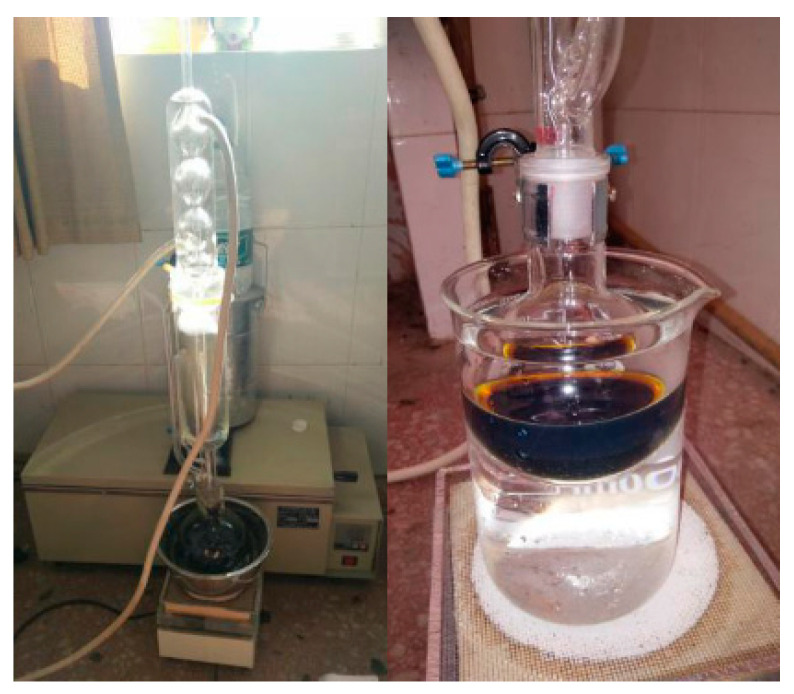
Purification of crumb rubber.

Crumb rubber is usually made by grinding waste tires and contains plasticizers and anti-aging agents, which will prevent rubber from being activated and need to be removed. The purification process of rubber powder was as follows: rubber was extracted with acetone in a Soxhlet extractor for 2 h (Figure 1), and then immersed in 5% NaOH for 1h, followed by being filtered and immersed in 10% HCl for 1 h. Finally the rubber powder was dried in an oven. 

The preparation of coating activated rubber was as follows: Firstly, polyamide 6 and formic acid (mass ration was 1:10) were put in a beaker and placed for 2 h at room temperature to obtain homogeneous solution. Then the purified rubber was added into formic acid solution of polyamide 6 and the mixture was stirred for 1 h at room temperature. Finally the mixture was filtered and dried in an oven to remove formic acid.

The preparation of grafting activated rubber was as follows: Firstly, 100 g purified rubber, 40 g acrylamide and 2.5 g potassium persulfate were mixed in 1000 mL water. Then the mixture was stirred for 4 h at 80 °C, followed by being washed with water and dried in an oven.

### 2.3. The Preparation of Rubber-Modified Asphalt Binder

Based on our previous research on optimization of the preparation technology [33], rubber-modified asphalt binder in this study was prepared as follows: 90 # base asphalt binder was heated to 140–160 °C and maintained for 1 h in an oven, and then crumb rubber was added. The mixture was stirred for 20 min manually and then sheared with high-speed shear instrument at 180 °C for 1 h. Unactivated rubber powder, coating-activated rubber powder and grafting-activated rubber powder were used as asphalt binder modifier, respectively, and the content was 20%. To ensure comparability, the base asphalt binder was also treated through the same process and recorded as A. Asphalt binder modified with unactivated rubber, asphalt binder modified with coating-activated rubber and asphalt binder modified with grafting-activated rubber were recorded as B, C and D, respectively (shown in Table 3).

### 2.4. Test Methods

#### 2.4.1. Characterization

The morphology of samples was observed by SEM (S-3000N, Hitachi, Tokyo, Japan). The FTIR spectra of rubber and modified asphalt binder were recorded through FTIR (Fourier-transform infrared spectroscopy) spectroscopy (Nicolet 380, Thermo Scientific, Waltham, MA, USA) with the wavenumber ranged from 400 cm^−1^ to 4000 cm^−1^.

#### 2.4.2. Conventional Physical Properties

The conventional physical properties of asphalt binder, such as penetration, softening point, ductility and viscosity, were tested according to the Chinese standard (JTG E 20-2011).

#### 2.4.3. Rheological Properties

The rheological characteristics of asphalt binder at high temperature was evaluated using a dynamic shear rheometer (DSR, MCR 302, Anton-Parr) according to JTG-T 0628-2011. Test were performed by parallel plates with a diameter of 25 mm and a spacing of 1 mm under strain controlling mode at fixed frequency of 10 rad/s. The temperature scanning range was from 58–88 °C.

The low temperature stiffness of the binders was tested using the bending beam rheometer (BBR) (TE-BBR, Cannon, Tokyo, Japan) according to JTG-T 0627-2011. The samples were rectangular with 125 mm × 12.7 m × 6.35 mm and loaded with 0.98 N for 4 min.

#### 2.4.4. Storage Stability

According to separation test regulations of polymer-modified asphalt binder (JTG-T 0661-2011), asphalt binder sample was injected into aluminium tube with diameter 25 mm, height 140 mm and sealed. The aluminium tube was then vertically placed in the oven at 163 °C and maintained for 48 h. Finally, the aluminium tube was taken out and frozen for 4 h at −4 °C and then equally cut into 3 sections. The softening point and rheological characteristics at high temperature of the top and bottom samples were measured, respectively.

## 3. Results and Discussion

### 3.1. Characterization of Activated Crumb Rubber

To investigate the chemical structure change of crumb rubber, an infrared spectroscopy test was conducted, and the spectra are shown in Figure 1, where UR represents unactivated rubber, CR represents coating-activated rubber with polyamide 6 and GR represents grafting-activated rubber with acrylamide. 

**Figure 2:**
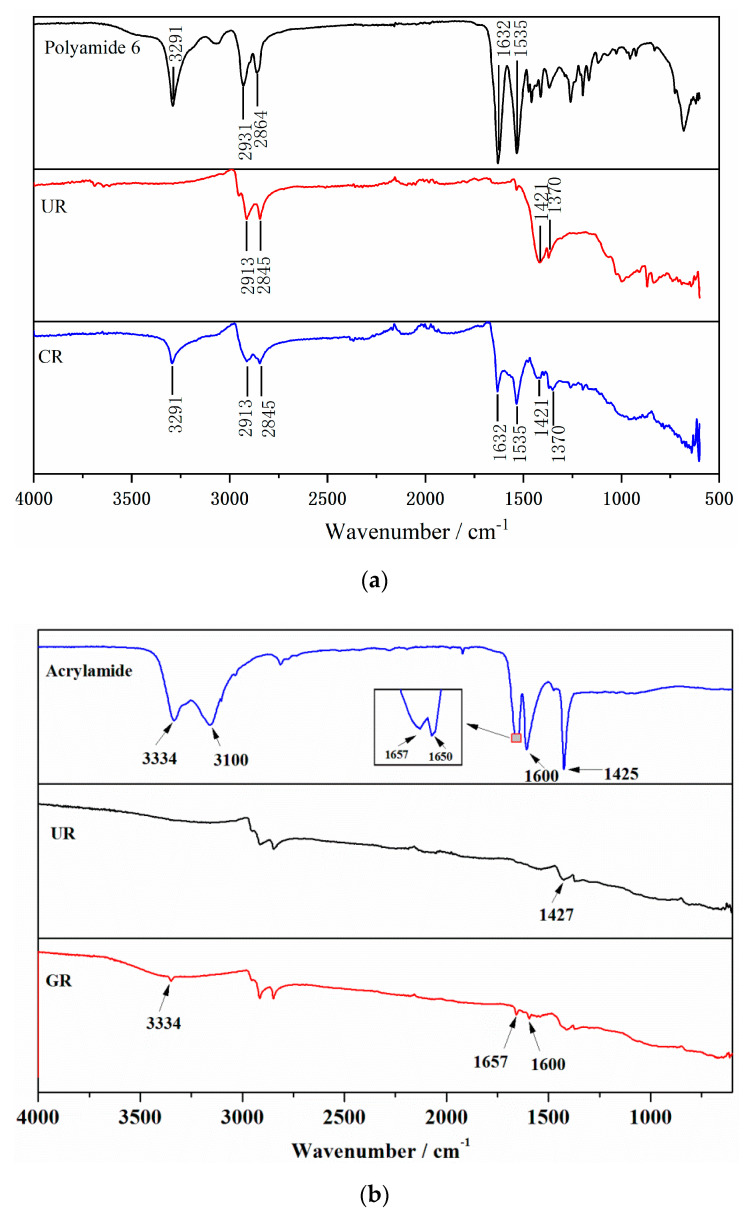
Fourier transform infrared (FTIR) spectra of crumb rubber. (**a**) spectra of polyamide 6, UR and CR; (**b**) spectra of acrylamide, UR and GR.

From Figure 2a, the main characteristic absorption peaks of polyamide 6 can be observed. Among of them, the peak of 3291 cm^−1^ is caused by the stretching vibration of N–H, the peaks of 3064 cm^−1^, 2931 cm^−1^, and 2864 cm^−1^ are ascribed to symmetrical and asymmetrical stretching vibration of saturated C–H, the peak of 1632 cm^−1^ is belong to the stretching vibration of carbonyl C=O, and the peak of 1535 cm^−1^ is assigned to the characteristic absorption of C–N. In the spectrum of UR, the peaks of 2913 cm^−1^ and 2845 cm^−1^ arose from the stretching vibration of –CH3 and antisymmetric stretching vibration of –CH2–, respectively, the peak of 1421 cm^−1^ is the antisymmetric angular vibration of –CH2–, and the peak of 1370 cm^−1^ is the in-plane bending vibration of –CH2–. The peaks of spectrum of CR are basically the superposition of polyamide and UR without new peak appearing, which means the coating activation of rubber with polyamide is a physical process and no chemical reaction occurs. According to the related literature of natural rubber graft copolymerization, the specific reaction process is as follows:

(1) The initiator decomposes into free radicals:
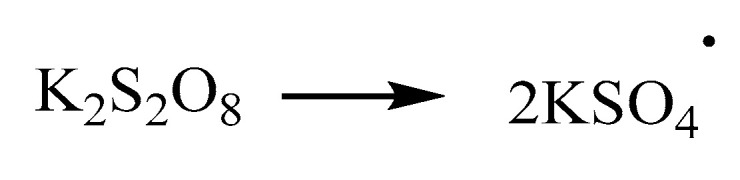
(2) Rubber hydrocarbon free radical (R.) initiated by free radicals:
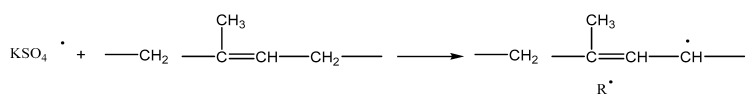
(3) Graft reaction:
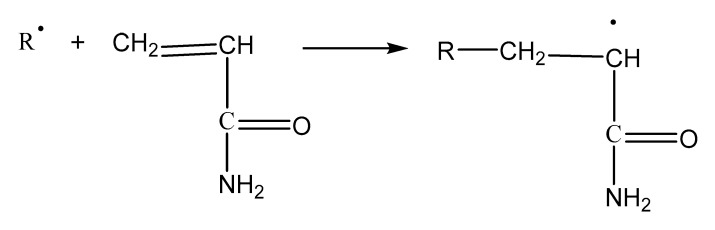

The infrared absorption spectra of acrylamide, UR and GR are shown in Figure 2b. In the curve of acrylamide, the peak due to the stretching vibration of –NH_2_ is found at 3334 cm^−1^ [34], and the peaks caused by the stretching vibration of =CH_2_ and C=C are observed at 3100 cm^−1^ and 1650 cm^−1^, respectively [35]. The peaks of 1657 cm^−1^ and 1600 cm^−1^ result from the –NHCO– stretching vibration [36]. The peak at 1425 cm^−1^ represents the stretching vibration of —CN. Compared with UR, there are three more absorption peaks of 3334 cm^−1^, 1657 cm^−1^ and 1600 cm^−1^ appearing in the spectrum of GR, which are derived from acrylamide. The peak at 1425 cm^−1^ ascribing to the stretching vibration of –CN overlaps with the peak at 1427 cm^−1^ due to the anti-symmetric angular vibration of –CH_2_– in rubber. However, the peaks of 3100 cm^−1^ and 1650 cm^−1^ do not show up, which indicates that chemical reaction occurs during the grafting activation of rubber.

Surface morphology of rubber powder has large effect on the performance of modified asphalt binder. The rubber powder produced by the low-temperature grinding method has smooth surface and sharp edges, while the rubber powder produced by ordinary temperature grinding method has rough surface with many pores [37]. In contrast, the latter has larger specific surface area, and can form stronger bonding force between rubber powder and asphalt binder, which gives modified asphalt binder better resistance to deformation [38]. Moreover, the elastic properties of the asphalt binder mixture is greatly effected by the morphological characteristics of the rubber particles, the larger the porosity and the rougher the surface of rubber, the better the elasticity of asphalt binder mixture.

**Figure 3:**
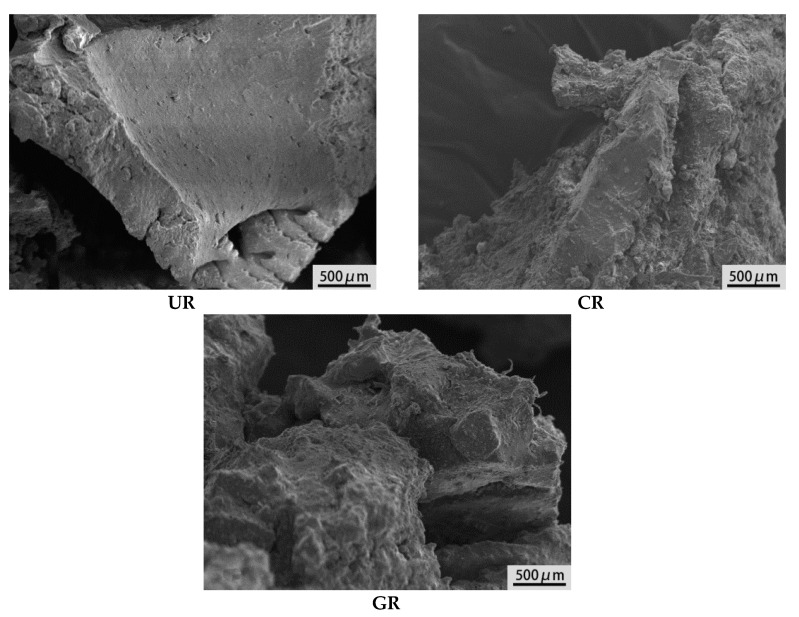
Scanning electron microscope (SEM) images of rubber.

Figure 3 shows the SEM images of UR, CR and GR. It can be seen that the surface of UR is smooth and flat, which is not conducive to the dispersion of rubber powder in asphalt binder, nor can promote rubber to combined with asphalt binder well [39]. By contrast, GR has the roughest surface and highest porosity, which expands the contact area and enhances the interaction between rubber and asphalt binder. Meanwhile, roughness and porosity of CR is between UR and GR.

### 3.2. Characterization of Modified Asphalt Binder

For conventional rubber powder-modified asphalt binder, the interaction between rubber powder and base asphalt binder is a key factor affecting the performance of modified asphalt binder. The effect of rubber powder activation on the interaction was explored through FTIR and SEM.

**Figure 4:**
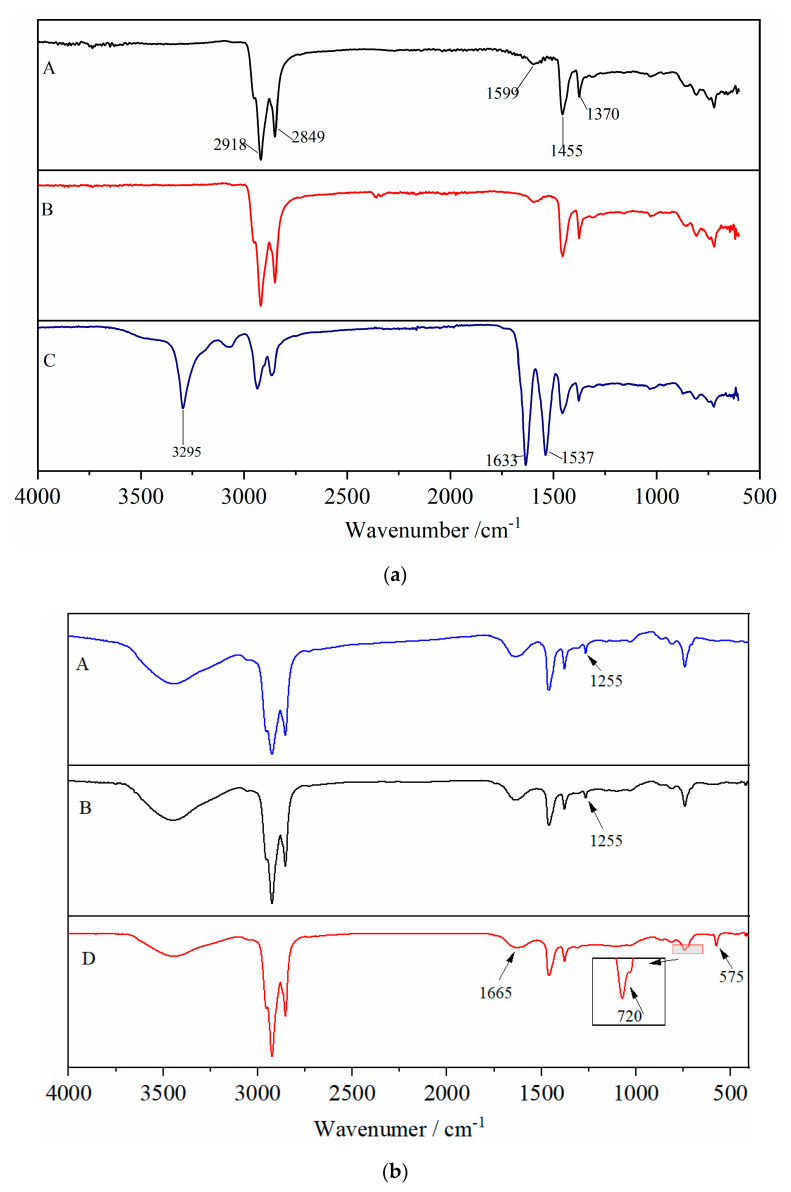
FTIR spectra of asphalt binders. (**a**) A—base asphalt binder, B—modified asphalt binder with unactivated rubber and C—modified asphalt binder with coating-activated rubber. (**b**) D—modified asphalt binder with grafting-activated rubber.

Figure 4a shows the chemical structure difference among base asphalt binder (A), modified asphalt binder with unactivated rubber (B), and modified asphalt binder with coating-activated rubber (C). The absorption peaks of base asphalt binder (A) are mainly in the range of 2800~3000 cm^−1^ and 1300~1700 cm^−1^, where the peak of 2849 cm^−1^ is ascribed to bending vibration of C–H, the peak of 2918 cm^−1^ is caused by the stretching vibration of –CH_2_–, the peak of 1599 cm^−1^ is belong to the stretching vibration of conjugated double bond C=C, the bending vibration of –CH_2_– is found at the peak of 1455 cm^−1^, and the symmetric bending vibration of –CH_3_ is observed at the peak of 1370 cm^−1^. In addition to the absorption peak of base asphalt binder, the other peaks appearing in B and C are corresponding to UR and CR, respectively, and there are no new peaks observed. The results of FTIR shows that no chemical reaction occur in the preparation of A, B and C, and the rubber powder swells in the base asphalt binder.

The spectra of A, B and D are presented in Figure 4b. In the curve of D, the peak of 575 cm^−1^ is ascribed to bending vibration of N–C=O of common amide. Both the peak at 1655 cm^−1^ caused by stretching vibration of C=O and the peak of 720 cm^−1^ generated by bending vibration of O–C=O belong to –COOH. The peak of 1255 cm^−1^ due to anhydride can be observed in both spectra of A and B, but it disappears in the spectrum of D. The possible reason is that anhydride group is very reactive and reacts with amino group during the preparation of D.

**Figure 5:**
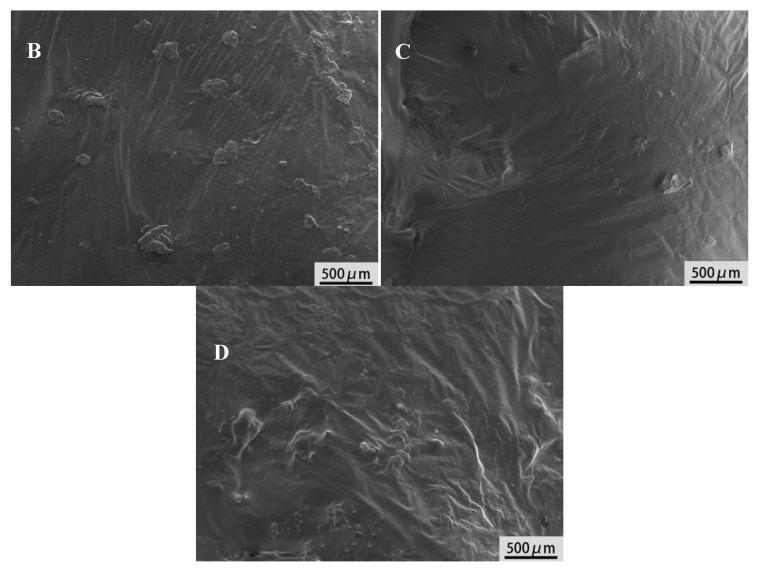
SEM images of modified asphalt binders. B—modified asphalt binder with unactivated rubber, C—modified asphalt binder with coating-activated rubber and D—modified asphalt binder with grafting-activated rubber.

The SEM images can intuitively reflect the compatibility of rubber powder and base asphalt binder, as shown Figure 5. It can be seen that the swelling degree of UR in base asphalt binder is lowest, and there is a clear interface between rubber powder and asphalt binder, indicating poor compatibility of them [40]. After being coating-activated, the visible rubber particles decrease and the compatibility is improved. It is worth noting that there is almost no rubber particles observed and the interface between rubber powder and asphalt binder is blurred in D. So the coating activation of rubber with polyamide 6 can increase the compatibility between rubber and asphalt binder due to the strong interaction between basic amide groups of polyamide and acid groups of asphalt binder. But the grafting activation of rubber with acrylamide has better effect because of the chemical reaction between amino groups of acrylamide and acid groups of asphalt binder [41].

### 3.3. Conventional Physical Properties of Modified Asphalt Binder

The conventional physical parameters of A, B, C and D are listed in Table 4. The addition of rubber powder can decrease the penetration and increase the softening point and viscosity of modified asphalt binder, especially when the rubber is activated before use. Rubber powder absorbs lightweight components in asphalt binder and swell, which results in the increase of viscosity and the reduction of penetration. This trend is more obvious as the swelling degree of rubber powder increases, so C has smaller penetration and bigger softening point and viscosity than B. There is both physical swelling and chemical reaction between GR and base asphalt binder, thus the effect of GR on the above parameters is the biggest. However, when it comes to ductility, the impact of rubber powder type is not regular. The addition of UR and CR both decrease this. This may be due to the low interaction between rubber and asphalt, and stress concentration generated by insufficiently swelled particles in modified asphalt binder during the stretching process, which causes the sample to break easily. However, the reaction between GR and asphalt binder improves the swelling degree of rubber and the interaction between rubber and asphalt binder which improves the flexibility of modified asphalt binder. Therefore, the ductility of D is close to A.

### 3.4. Rheological Properties of Modified Asphalt Binder

The rheological properties of modified asphalt binder at high temperature with loading frequency at 58 °C, 64 °C, 70 °C, 76 °C, 82 °C, 88 °C were measured by DSR. The complex shear modulus G*, and phase angle δ can effectively reflect the deformation resistance and viscoelasticity of asphalt binder binder as important rheological parameters.

**Figure 6:**
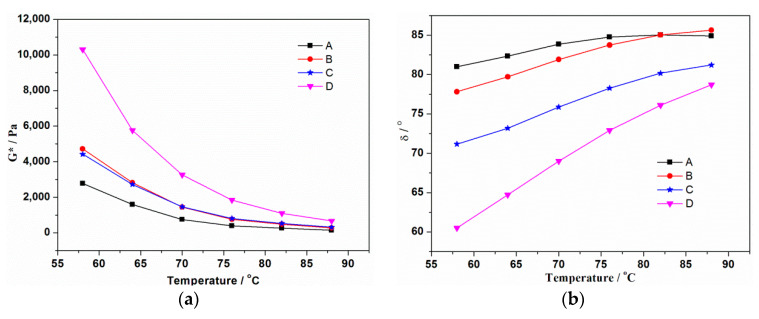

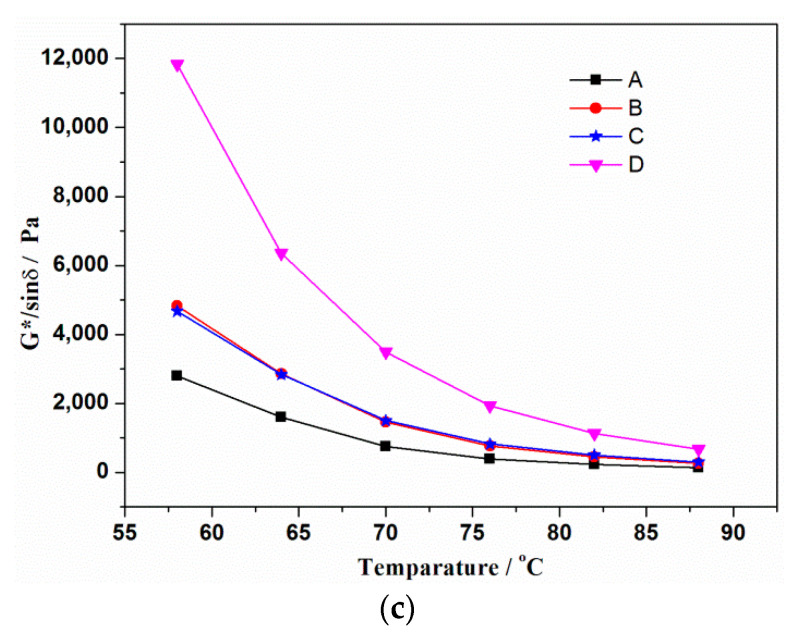
(**a**) G*, (**b**) δ and G*/sinδ, (**c**) of asphalt binders.

In Figure 6a, G* of all tested asphalt binder samples decreases with temperature. At low temperature, the elastic components in rubber play a dominant role, but asphalt binder shows non-Newtonian viscous fluid performance at high temperature, so the higher the temperature, the worse the deformation resistance [4]. The average value of G* of B increased by 70.4%, 77.4%, 92.8%, 93.4%, 90.2% and 86.6% compared with A at 58 °C, 64 °C, 70 °C, 76 °C, 82 °C, 88 °C, respectively, which means that the addition of rubber is beneficial to improve the deformation resistance. The G* of B and C are almost the same except at low temperature, indicating that coating activation of rubber has little effect on the deformation resistance. But grafting activation of rubber remarkably improves the G* of asphalt binder, which can be attributed to the chemical reaction and strong interaction between rubber and asphalt binder in D.

Figure 6b shows the phase angle results of all test samples. Phase angle is the time lag of the applied stress and the resulting strain, which reflects the viscoelasticity of the asphalt binder. It can be seen that A has the highest δ value, then B followed by C, and D has the lowest δ value, so the order of the proportions of elastic components is just the opposite.

The rutting factor calculated from G*/sinδ characterizes the anti-rutting ability of asphalt binder; the larger the G*/sinδ value, the better the anti-rutting ability. Figure 6c shows the variation trend of G*/sinδ of four types of asphalt binder as a function of temperature. Due to the δ values of all samples are in the range of 60~90°, so the curves of G*/sinδ are similar to the curves of G*. Overall, D has the best anti-rutting ability, then C, followed by B, and finally A.

The low-temperature creep performance of the four types of asphalt binder was measured using BBR, creep stiffness S and creep rate m were obtained as important evaluation indexes. S represents the toughness, the larger the S value, the more brittle the asphalt binder material and the easier the road surface can crack. Creep rate m reflects the stress relaxation ability, the larger the m value, the better the stress relaxation ability of the asphalt binder material, and the lower the possibility of low-temperature cracking.

The results of the BBR test are listed in Table 5. For all asphalt binders tested, S increases and m decreases with a reduction of the temperature, which indicates that all the samples have the tendency to become hard and brittle when temperature declines. After being modified with rubber, the S of asphalt binder increases and m decreases. This is mainly due to the fact that as an elastic material, rubber has less stiffness at low temperature and can improve the tenacity of asphalt binder. However, compared with asphalt binder modified with UR, the low temperature of asphalt binder modified with coating-activated rubber decreases slightly. This is because the elasticity of polyamide is poor, which restrains the effect of elasticity of rubber [42]. Except for the elasticity of the rubber powder itself, the interaction between rubber and asphalt binder also has an important effect on the creep performance. A chemical reaction occurring in the preparation of modified asphalt binder with GR greatly enhances this interaction, so D has the best low temperature performance. C is the second, B is the third and A is the last in order of low-temperature performance. In addition, the evaluation of low temperature performance from the perspective of ductility and BBR is inconsistent. In the case of vehicle load, the low-temperature performance of the asphalt pavement is related to the maximum tensile stress that can be withstood and has little relationship with the stretchable length [43]. Therefore, using ductility to evaluate the low-temperature performance of asphalt binder has certain limitations. The bending beam creep test is based on rheology, using the bending beam creep principle to measure the deflection change with time, and the low-temperature performance is characterized by the stiffness modulus and creep rate. Therefore, it is considered that the results of the BBR test are more relevant to the low-temperature performance.

### 3.5. Storage Stability of Modified Asphalt Binder

Asphalt binder modified with rubber has a tendency to separate into a rubber-rich phase (rubber swelled by maltene molecules) and an asphalt binder-rich phase [44], especially during storage at high temperature, which is one of the key problems restricting the large-scale application of rubber powder modified asphalt binder. A large difference in density and weak interaction between are the main reasons of segregation. Introducing functional groups that can interact strongly with asphalt binder to rubber through activation treatment is expected to improve the storage stability in this study.

**Figure 7:**
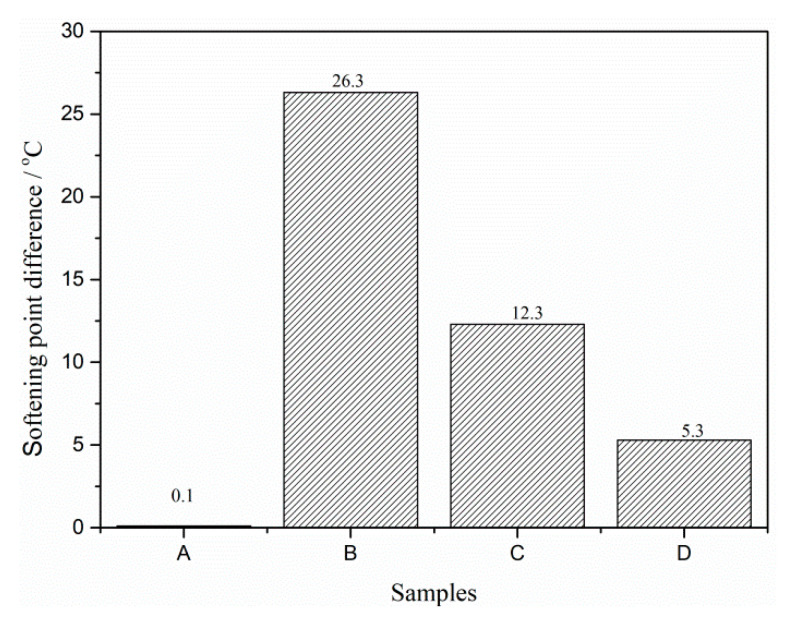
Softening point difference of asphalt binders.

Figure 7. shows the softening difference of asphalt binders A, B, C and D. After being stored at 163 °C for 48 h, the softening difference between the top sample and bottom sample of base asphalt binder (A) is only 0.1 °C, which means that base asphalt binder is very stable during storage and there is almost no segregation. The softening difference of B is 26.3 °C and is the highest of these four types of asphalt binder. When rubber activated by polyamide coating is used as modifier, the softening difference decreases to 12.3 °C, which is 46.8% of B. It proves that polyamide coated on rubber promotes the interaction between rubber and asphalt binder. D has the lowest softening difference of 5.3 °C, only 20.2% of B, presenting the dramatic reduction of segregation. That is because the acrylamide grafted on the rubber not only improves its swelling degree but also reacts with the acid groups of the asphalt binder.

**Figure 8:**
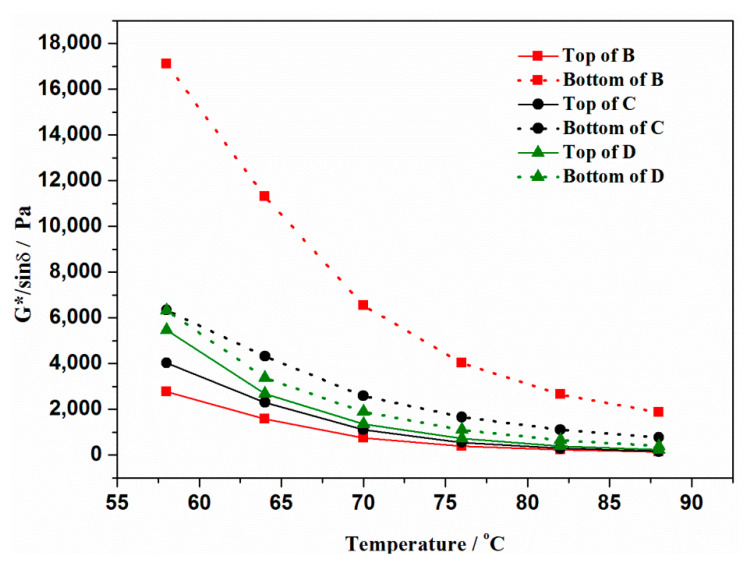
G*/sinδ of top and bottom samples of modified asphalt binder.

To further study the effect of the activation methods of crumb rubber on the storage stability and compatibility of modified asphalt binder, the rheological properties were tested via DSR. The rutting factors of samples at the top and bottom are shown in Figure 8. The bottom samples of all asphalt binder binders have larger rutting factor than the top samples, but the difference between bottom and top is obviously influenced by the activation of rubber. The bottom sample of B has the largest rutting factor, and the top sample has the smallest. This can be explained by the fact that inactivated rubber particles accumulate at the bottom due to bigger density than asphalt binder and results in serious segregation. The rutting factor difference of D is the smallest and that of C is between B and D.

Meanwhile, the segregation index (SI) obtained from DSR test results also can be used to evaluated the storage stability [45]. SI can be calculated according to the following formula:

(1) $$\mathrm{SI}={\left(G^{*}/\sin\delta \right)}_{\mathrm{bottom}}/{\left(G^{*}/\sin\delta \right)}_{\mathrm{top}}$$

where $\mathrm{SI}={\left(G^{*}/\sin\delta \right)}_{\mathrm{bottom}}/{\left(G^{*}/\sin\delta \right)}_{\mathrm{top}}$ are the rutting factor of the bottom and top samples of tube, respectively. The closer to 1.0 the SI, the smaller the segregation degree.

**Figure 9:**
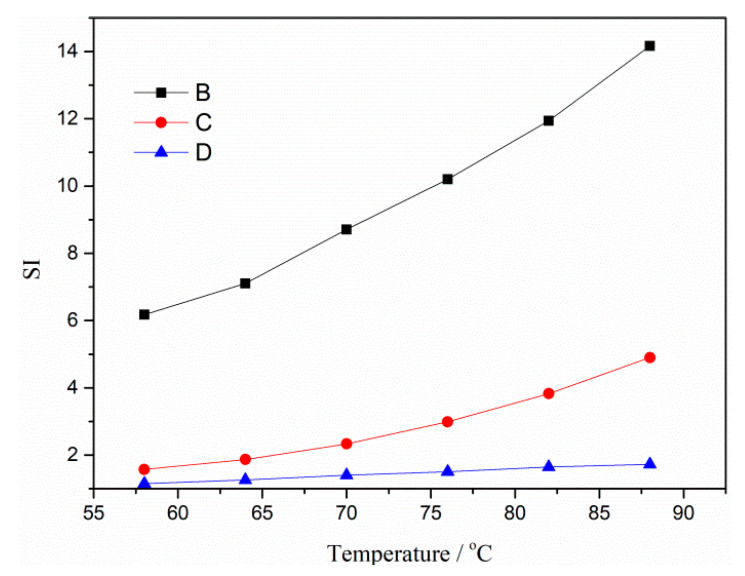
Segregation index (SI) of modified asphalt binder.

From Figure 9 one can see that B has the biggest SI values within the temperature scan range, followed by C, and D has the value closest to 1. This result once again proves that the activation of crumb rubber can improve the storage stability of modified asphalt binder, and grafting activation with acrylamide has a better effect compared with coating activation with polyamide 6.

### 3.6. Modification Mechanism of Asphalt Rubber

**Figure 10:**
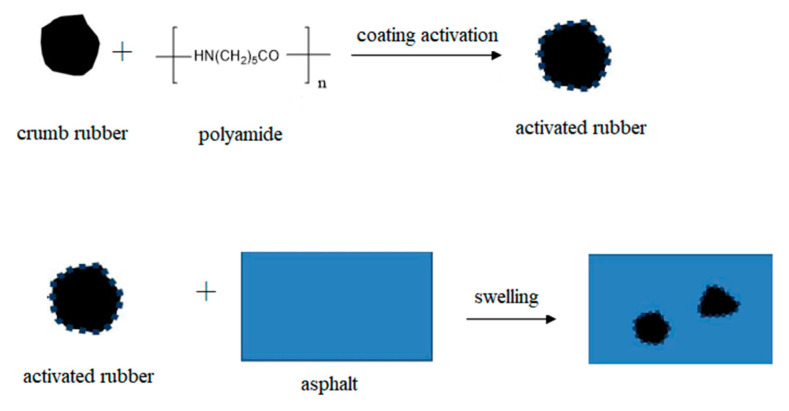
Schematic diagram of the modification mechanism with CR (coating-activated rubber with polyamide 6).

Based on the above results and discussion, the modification mechanism of asphalt rubber is explored by combining microstructure and macroscopic performance. Before activation, the surface of rubber is dense and smooth, so it cannot swell adequately in asphalt binder and has weak interaction with asphalt binder. However, after being coating activated with polyamide 6, the surface becomes looser and rougher, which improves the specific surface area and surface activity of rubber. From the FTIR results, no chemical reaction occurs during the preparation of C. Although rubber is still mainly swelled in asphalt binder, the interaction between rubber and asphalt binder is strengthened, and thus the high-temperature performance and storage stability is increased, and the schematic diagram of the modification mechanism is shown in Figure 10. For rubber activated by grafting with acrylamide, chemical reactions generate during both the activation of rubber and the preparation of modified asphalt binder. According to the structure analysis and chemical reaction principle, it is inferred that the chemical reaction between amino groups in grafted rubber and acid groups in asphalt binder occurs as follows.

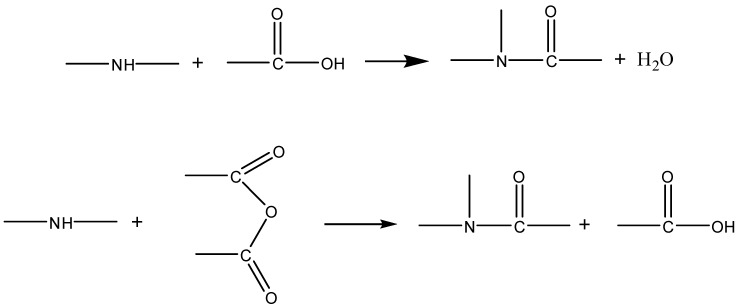


**Figure 11:**
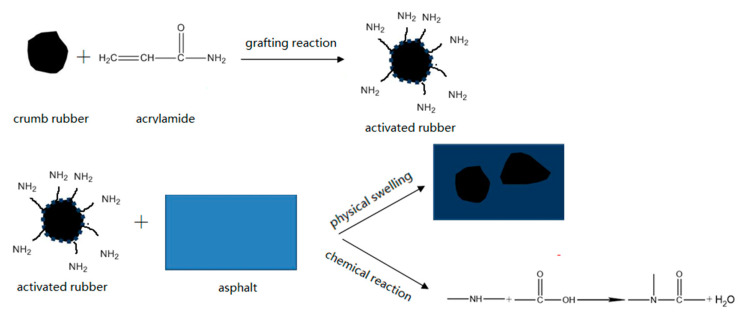
Schematic diagram of the modification mechanism with GR (grafting-activated rubber with acrylamide).

Thus there is not only physical swelling but also chemical action between GR and asphalt binder, which gives the modified asphalt binder relatively stable structure and effectively improves its storage stability, and the schematic diagram of the modification mechanism is shown in Figure 11.

## 4. Conclusions

In order to enhance the interaction between the crumb and asphalt and thereby improve the performance of modified asphalt binder, two activation methods were adopted to treat crumb rubber before it was used to modify asphalt binder. Four types of asphalt binder were prepared through the same process, and their properties were measured to investigate the effect of activation of rubber. The main conclusions are summarized as follows:

(1) After being activated, the surface of rubber is porous and rough. Coating activation of rubber with polyamide 6 is a physical process, but chemical reactions occur during grafting activation of rubber with acrylamide.
(2) The penetration, softening point and viscosity results show that the activation of rubber can increase the high temperature properties of modified asphalt binder, and the grafting activation is superior to the coating activation.
(3) All the three types of rubber decrease the ductility of the modified asphalt binder, especially coating-activated rubber. However, according to the results of BBR, the addition of rubber can improves the low temperature, and grafting activated rubber has the largest effect.
(4) Based on the DSR results, it is found that the addition of rubber is beneficial to improving the deformation resistance and rutting factor. The coating activation of rubber has little effect on high temperature rheological properties of asphalt binder. However, grafting activation of rubber has a remarkable effect, which can be attributed to chemical reaction and strong interaction between rubber and asphalt binder.
(5) The storage stability of modified asphalt binder was evaluated by polymer segregation and DSR test. The softening difference of B, C and D were 26.3 °C, 12.3 °C and 5.3 °C, respectively. It proves that both rubber activation methods can promote the interaction between rubber and asphalt binder, and then improve the storage stability of modified asphalt binder.
(6) The modification mechanism of asphalt binder rubber was explored combining microstructure and macroscopic performance. The interaction between inactivated rubber, coating-activated rubber and asphalt binder is mainly swelling. However, there is both physical swelling and chemical action between grafting rubber and asphalt binder.

## Figures and Tables

**Table 1:** Technical parameters of base asphalt binder.

Item	Units	Test Results	Standard
Penetration (25 °C, 100 g, 5 s)	0.1 mm	87	JTG-T0604-2011
Softening temperature	°C	47	JTG-T0606-2011
Ductility (10 °C, 5 cm/min)	cm	>100	JTG-T0605-2011
Kinematic viscosity (135 °C)	Pa s	147.3	JTG-T0625-2011
Density	g/cm^3^	1.009	JTG-T0603-2011
RTFO treated at 163 °C for 85 min			
Quality change	%	−0.063	JTG-T0610-1-2011
Residual penetration ratio (25 °C)	%	59.5	JTG-T0610-2-2011
Residual ductility (10 °C)	cm	3.5	JTG-T0605-2011

RTFO-(Rolling Thin Film Oven) aging.

**Table 2:** Technical parameters of crumb rubber.

Item	Result	Standard
Water content (%)	1.1	HG/TXXX-2001 7.2.2
Ash content (%)	8.9	GB4498
Acetone extract content (%)	13.1	GB/T3516
Density (g/cm^3^)	1.14	GB/T533
Tensile strength (MPa)	15	GB/T528
Elongation at break (%)	650	GB/T52

**Table 3:** Samples of asphalt binder.

Sample	Modifier	Content of Modifier (%)
A	no	0
B	Unactivated rubber	20
C	Coating-activated rubber	20
D	Grafting-activated rubber	20

**Table 4:** The conventional physical parameters of asphalt binder.

Item	Sample				Standard
	A	B	C	D	
Penetration at 25 °C (mm)	78.1	57.3	55.2	50.9	JTG-T0604-2011
Softening Point (°C)	50	58.1	60.5	63.5	JTG-T0606-2011
Ductility at 5 °C (mm)	98.5	60.6	54.5	96.8	JTG-T0605-2011
Viscosity at 175 °C (mPa.s)	81	766	1124	1580	JTG-T0625-2011

**Table 5:** Creep stiffness modulus S and creep rate m value.

Simple	−12 °C		−18 °C		−24 °C	
	S (MPa)	m	S (MPa)	m	S (MPa)	m
A	131.0	0.360	325.0	0.305	615.0	0.230
B	75.6	0.396	99.0	0.320	297.3	0.296
C	79.0	0.365	110	0.315	295.0	0.251
D	67.3	0.422	63.4	0.368	143.3	0.309
